# Impacts of Stressors on Riparian Health Indicators in the Upper and Lower Indus River Basins in Pakistan

**DOI:** 10.3390/ijerph192013239

**Published:** 2022-10-14

**Authors:** Amin Hira, Muhammad Arif, Nowsherwan Zarif, Zarmina Gul, Xiangyue Liu, Yukun Cao

**Affiliations:** 1 Department of Forestry Economics & Management, Northeast Forestry University, Harbin 150040, China; 2 Biological Science Research Center, Academy for Advanced Interdisciplinary Studies, Southwest University, Chongqing 400715, China; 3 Pakistan Forest Institute, Peshawar 25000, Pakistan; 4 College of Chemistry, Chemical Engineering and Resource Utilization, Northeast Forestry University, Harbin 150040, China

**Keywords:** Terbela Dam Reservoir, Indus River, riparian health assessment, environmental indicators, rapid appraisal, ecosystem function, pressure indicators

## Abstract

Riparian zones along rivers and streams provide ecosystem services that may change over time as disturbances increase and deteriorate these buffer zones globally. The effect of stressors on ecosystem services along the rivers in underdeveloped countries is unclear, which impacts the environment directly in the form of riparian health indicators (RHIs). This study fills this gap and measures the impact of stressors on RHIs (parameters of habitat, plant cover, regeneration, exotics, and erosion) in the Indus River basin (IRB) in Pakistan. Data on 11 stressors and 27 RHIs were collected using a field-based approach in 269 transects in the upper and lower Indus basins (UIB and LIB) in 2020 and analyzed using multivariate statistical methods. The Kruskal–Wallis tests (*p* < 0.05) indicated that RHIs varied significantly under the influence of stressors in the UIB and LIB. However, their highest mean values were found in the UIB. Principal component analysis revealed the key RHIs and stressors, which explained 62.50% and 77.10% of the variance, respectively. The Pearson correlation showed that stressors had greater impacts on RHIs in LIB (with r ranging from −0.42 to 0.56). Our results also showed that stressors affected RHI indices with r ranging from −0.39 to 0.50 (on habitat), −0.36 to 0.46 (on plant cover), −0.34 to 0.35 (on regeneration), −0.34 to 0.56 (on erosion), and −0.42 to 0.23 (on exotics). Furthermore, it was confirmed by the agglomerative hierarchical cluster that indices and sub-indices of RHIs and stressors differ across the UIB and LIB. These findings may serve as guidance for managers of large rivers and ecosystem service providers to minimize the environmental impact of stressors in terms of RHIs.

## 1. Introduction

Human activities resulting from urbanization and industrialization have deteriorated the riparian zones of many countries [1,2,3,4]. It is a fact that riparian zones naturally improve water quality since they ensure the safety of both the river and the environment [5]. Streams and rivers supply 90% of the freshwater needed to sustain life in the biosphere, and they are the primary sources of water for reservoirs and lakes [6]. Natural ecological landscapes formed by river riparian zones are considered one of the world’s biodiversity hotspots [7]. Despite this, these buffer zones represent some of the most sensitive and irrecoverable natural ecosystems [8]. While rivers have both positive and negative global impacts, their riparian zones are in constant danger of deterioration due to changes in stressors [9]. Because land-use patterns have complex effects on riparian health, it is critical to understand the relationships between stressor variation and the riparian zone condition around rivers [4,10]. The sustainable use of water resources requires the investigation of this topic [11,12]. In particular, such research will tell us how changes in stressors might affect the riparian zones of large rivers such as the Indus River basin (IRB).

Land surfaces that are naturally used for agriculture and other purposes are continually converted into artificial ones to accommodate the growth of urban and industrial needs [13]. This leads to the weakening of marginal areas and the degradation of riverine areas [7,14]. Several types of activities directly alter the physical characteristics of the land and alter riparian ecosystems and their associated habitats [15]. Riparian zones are often permanently affected by land use that has higher levels of human activity [16]. These activities include residential, industrial, commercial, and transportation activities. The relationship between artificial land use and riparian ecosystems is negatively correlated in earlier quantitative studies [17,18]. Nonetheless, the relationship between riparian health conditions and stressors is somewhat more complex [19]. Previous studies have demonstrated the effect of land-use changes and adverse environmental events on the vegetation in buffer zones, which in turn affects riparian health and rapidly alters the area’s physical characteristics [20]. Different scenarios of land use have been studied in various parts of the world to assess the impacts of riparian zones [7,13]. It is rare in the literature to find studies that evaluate the response of riparian zones to stressors under different basins of large rivers, such as the IRB.

However, the effects of stressors in buffer zones may vary depending on the purposes for which they are utilized [21]. Rural areas are significantly affected by agricultural activities, while urban areas are significantly impacted by concrete structures [22]. When researchers compared the correlations between near-field events and far-field events, they found that the correlations between near-field events were stronger, which suggests that the stressor was more concentrated in the near-field events [2,23]. For this reason, it is extremely relevant to investigate how riparian zones respond to different stressors in different river basins. This step will ensure that these ecosystems are conserved and managed sustainably.

The land use and management practices of rivers significantly impact watershed protection strategies [1]. Globally, there is a more pressing need for effective policies than in the past century. The growing population is the driving force behind substantial developments in every service industry sector to meet the public’s needs [24]. The status change of water bodies is largely due to this desire. As a result, the area reduction ratio for these water bodies has been particularly high even in developing countries, like in China, where it has increased from 135 to 8700 km^2^ since the 1970s, despite the continuous implementation of water conservation measures at the national level by the Chinese government to ensure that water resources are utilized to their full potential [2,6]. Similarly, rivers in Pakistan are among the most threatened ecosystems due to long-standing human settlements along their riverbanks. Their riparian zones are vulnerable to prolonged disturbances because of a long history of human settlement there [25]. Large river basins can have long periods of water retention, which can lead to the rapid deterioration of riparian ecosystems and biodiversity [26]. The Indus River, the primary water source for agriculture in Pakistan, is extremely fragile compared to other rivers [27]. It has been noted in previous reports that this river faces several major pressures and is considered one of the most affected rivers in the world regarding sediment loads [28,29]. Various environmental and physical factors have altered the basin pattern of the Indus River because of human disturbance [30,31].

This study focuses mainly on the Indus River flowing into the IRB, the largest river in Pakistan [25]. It is the main source of freshwater in Pakistan and the lifeline of the country [30]. Because of different geomorphology and geographic location, the riparian zones of the IRB are very vulnerable, which is particularly relevant for the purposes of environmental and ecological protection [29,30]. It is evident from the literature that the river ecosystem can be damaged in terms of habitat, plant cover, regeneration, exotic species, and erosion [25,29,31]. Once it becomes damaged, it is challenging to restore it to its original state [32]. Several riparian health indicators (RHIs) have been declining in the IRB, and the situation has become more problematic because of recent variations in stressors. By merely looking at one source alone, it is impossible to determine the pressure effects in the river basin [1,11]. There is a need for statistical models and theoretical studies to generate technical information on the effects of different river basins on riparian areas regionally and globally [2,15]. This study assessed changes in the riparian zones of the Indus River under the influence of stressors originating from the river’s different basins (i.e., the upper Indus basin (IUB) and the lower Indus basin (LIB)). More specifically, we (1) examined the variation between indices and sub-indices of RHIs and stressors observed in the UIB and LIB areas; (2) identified the key indicators responsible from RHIs and stressors for this variation in the overall riparian zone; (3) measured the correlation between stressors and RHIs and measured the degree to which variables interact with one another in different basins; and (4) classified the statistical similarities between indices and sub-indices of RHIs and stressors within the UIB and LIB sites. It is hoped that the results of this research will help come up with ways to take care of riparian zones when different stressors are around.

## 2. Materials and Methods

### 2.1. Description of the Study Area

**Figure 1:**
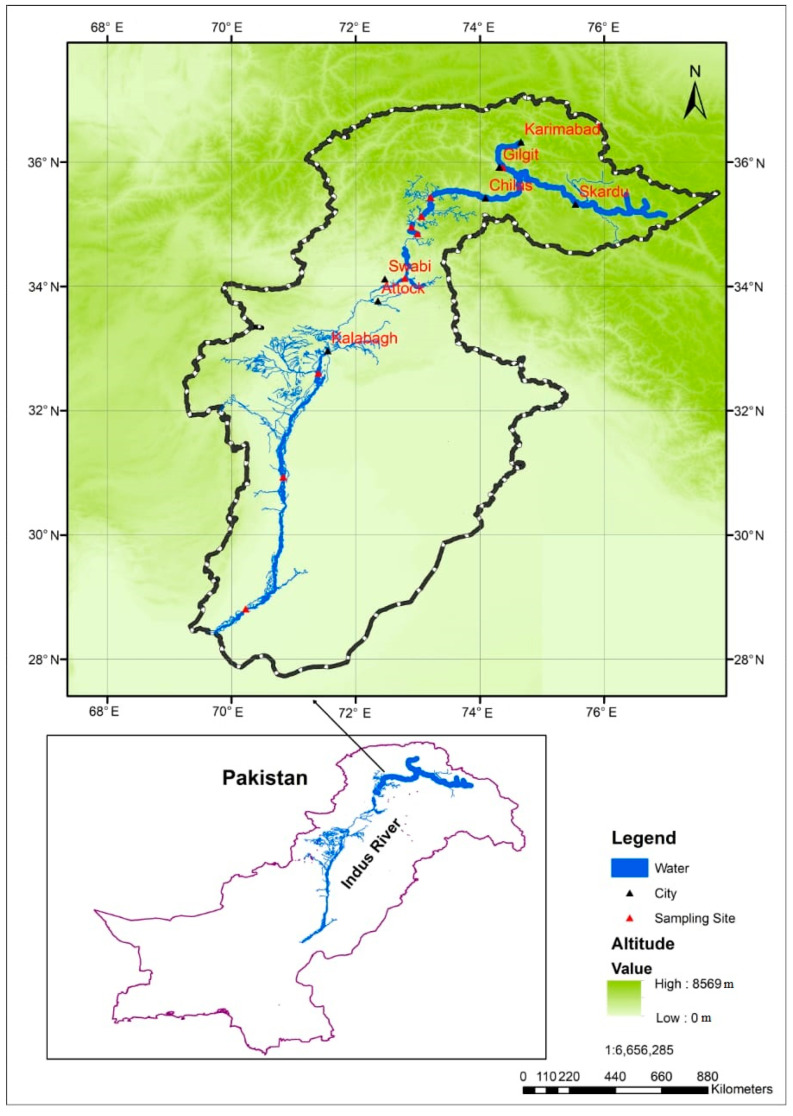
Location of sample sites in the upper and lower Indus basin riparian zones of the Indus River in Pakistan.

Among the five largest river basins in Pakistan (Indus, Chenab, Jhelum, Ravi, and Sutlej), the IRB is perhaps the most famous. It is situated between 29°30′ N~37°10′ N and 70°15′ E~77°0′ E (Figure 1) [25]. The area of the IRB is approximately 139,202 km^2^; 53%, 39%, and 8% of this area is located in Pakistan, India, and China, respectively [31]. There are several branches of this river that flow through India and Pakistan and empty into the Arabian Sea, which originates from the Tibetan Plateau’s Lake Mansarovar. Sediment is produced in large quantities by the Indus River. At the time of planning and design, the sediment load estimated by consultants at Tarbela Dam, which is at the intersection of the UIB and the LIB, was 242.4 mcm annually [27]. The Indus River meets other rivers in Pakistan at Kharmong and Panjnad [28]. UIB, which represents 54% of the IRB, and LIB, which represents 46% of the IRB, are two major components of the IRB because of geographical characteristics and land use patterns. It is important to remember that the LIB mentioned is the midstream part of the whole IRB. The UIB is fed primarily by westerly winds and glaciers at an altitude of 440 to 8361 m above sea level [31]. The elevation of LIBs varies between 70 and 440 m asl. In these regions, the climate tends to be arid or semi-arid, with the flow of water predominantly controlled by upstream discharge, tributaries, and monsoon rains. The UIB receives 200 to 1400 mm of precipitation per year while its temperature ranges from 5 to 20 °C. The LIB is generally characterized by temperatures ranging from 10 to 32 °C and precipitation ranging from 200 to 1200 mm [26,29]. In addition to water bodies and forests, shrublands, grasslands, agricultural and barren areas, built-up areas, as well as snow and glaciers, the IRB recognizes several major categories of land use [30]. Different geological formations produced different orders of dominant major cations between UIB and LIB. According to the suitability assessment, the river water is suitable for drinking in UIB. However, it did not meet the guidelines the World Health Organization set regarding irrigation in LIB [27]. This problem is primarily due to soil degradation. The Tarbela Dam Reservoir is located 65 km northwest of Islamabad along the Indus River. The reservoir can store 14,295 mcm of water at full capacity [25]. An estimated 76,274 mcm of water is received each year by the Tarbela Dam Reservoir [30]. It is estimated that the dam will remain in service for another 85 years [26]. The delta could advance towards the power intakes if sediment deposits accumulate in the reservoir, causing interference with outlet openings and other dam functions.

### 2.2. Field Investigation

**Figure 2:**
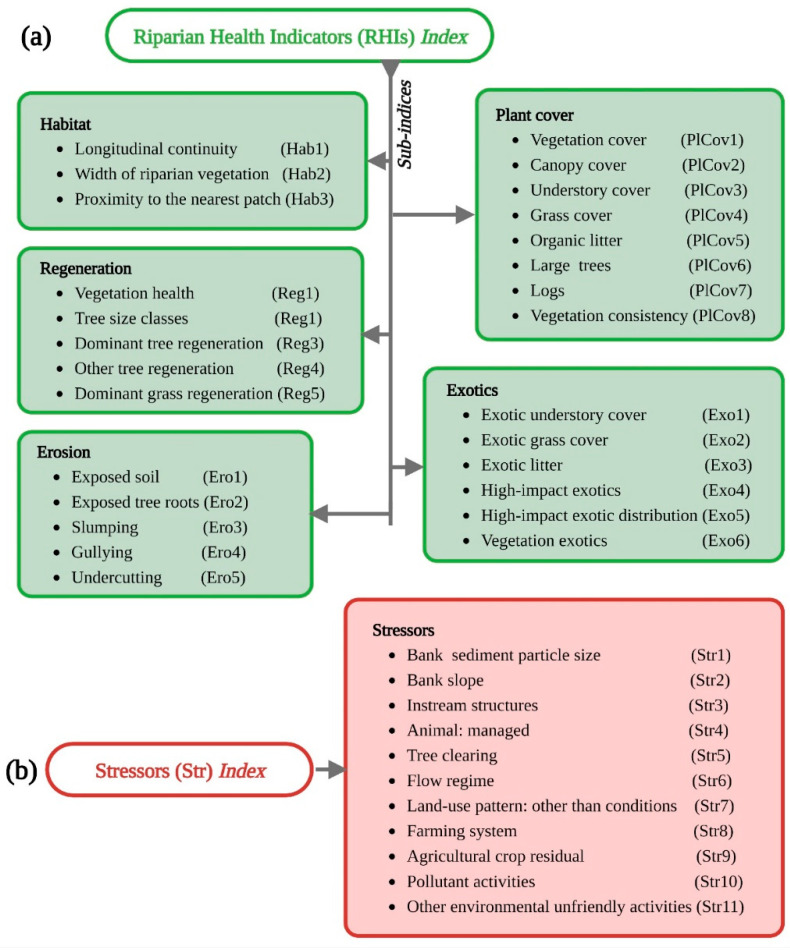
List of the riparian health indicators (**a**) and stressors (**b**), along with their respective abbreviations inside the parentheses, used for the upper and lower Indus basin riparian zones of the Indus River in Pakistan.

We collected data in 2020 from 269 transects (145 and 124 for UIB and LIB transects, respectively) within the IRB, considering their proportional contributions. This study aimed to obtain real-life data on RHIs and stressors by adopting an innovative approach from the literature (see Arif et al. [2,15,32]). The data collection methods for each parameter are described in various sources (e.g., literature, reports, and databases). This approach was introduced by Jansen et al. [33] to study the riparian zone in Australia, and since then, it has been adopted by other researchers [19,23,34]. This study included several indicators that were subject to constraints specific to the IRB. Thus, we used the method described by Lanzanova et al. [35] to estimate new indicators and to fill in missing measurements. Each component of the riparian zone was quantified by establishing a 100 m long, 20 m wide transect parallel to the Indus River (see details in Supplementary Materials). Indicators of the riparian zone were studied from two perspectives (see Figure 2). Indicators such as habitat, plant cover, regeneration, exotics, and erosion are included in the RHI category. The indicators in this study corresponded to the indicators observed in riparian zones during the study period. The second category consists of broad stressors. In order to present a viable representation of the entire IRB, the researchers incorporated the maximum number of indicators reported in the literature.

### 2.3. Statistical Analysis

We conducted the statistical analysis using Origin software, which was released in 2022 in Northampton, MA, USA. There were significant differences between RHIs and stressors in the UIB and LIB as determined by nonparametric Kruskal–Wallis tests. RHIs and stressor indices or sub-indices can be compared with this nonparametric test [2]. UIBs and LIBs relating to RHIs and stressors were also analyzed by principal component analysis (PCA) (e.g., factor analysis). Hence, leading factors are identified, reducing potential challenges when 38 indicators are considered [19]. We correlated and associated RHIs and stressors with UIB and LIB river networks based on Pearson correlation coefficients. This study employs correlation coefficients to represent the connection commonly found in linear models [8]. Hierarchical cluster analysis was then used to analyze the patterns in the UIB and LIB indexes, taking into account indicators of RHIs and stressors. The comprehensive analysis combined similar characteristics within complex river environments [32]. Furthermore, the analysis can be used to distinguish between the UIB river network and the LIB river network.

## 3. Results

### 3.1. Distribution of Riparian Zone Characteristics

**Figure 3:**
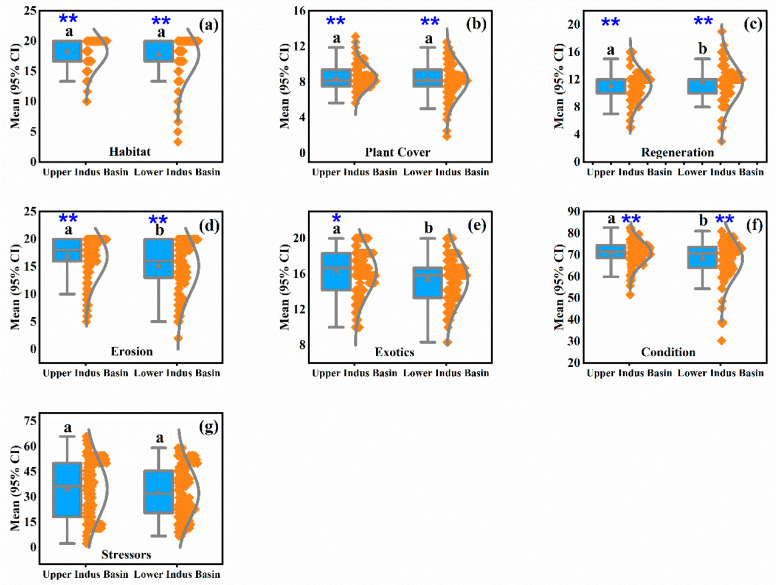
Box and whisker plots for riparian health indicators (**a**–**f**) and stressors (**g**) measured in the upper and lower Indus basin riparian zones of the Indus River in Pakistan. The *y-axis* denotes the mean scores with 95% confidence interval (CI). The *silver horizontal line* represents the median, and the *silver triangle* sign symbolizes the mean. The boxes represent the 25th–75th percentiles, and the whiskers outside the boxes represent the 10th–90th percentiles. The *silver curve* symbolizes the shape of data distribution, whereas the *orange rhombus* represents data distribution. Note: significance is at *p* < 0.01 (**) or *p* < 0.05 (*) of the Kruskal–Wallis tests. Moreover, pairwise comparisons between the upper and lower Indus basins are marked with non-identical lowercase letters (a,b) using Dunn-Bonferroni post hoc tests.

This study presents seven box and whisker plots (a–g) containing a 95% confidence interval that illustrates differences between the RHIs index (condition) and its sub-indices (habitat, plant cover, regeneration, erosion, exotics) and the stressor index measured in the riparian zones of both the UIB and LIB of the IRB (Figure 3). In terms of RHIs, UIB had the highest average score of 70.73% ± 5.64% (mean ± standard deviation) for overall condition, with 18.14% ± 2.71% for habitat, 8.57% ± 1.21% for plant cover, 16.78% ± 4.06% for erosion, and 16.37% ± 2.51% for exotics. The RHIs index and its sub-indices were statistically significant for UIB indicators at *p* < 0.01** and *p* < 0.05* for exotics only. The mean score of the regeneration sub-index was relatively higher in the LIB (11.37% ± 2.89%), but it was statistically insignificant at *p* < 0.05*. The stressor index was also statistically insignificant at *p* < 0.05*, with mean scores of 35.00% ± 17.17% for the IRB’s UIB.

### 3.2. Selection of the Principal Factors by Factor Analysis

**Figure 4:**
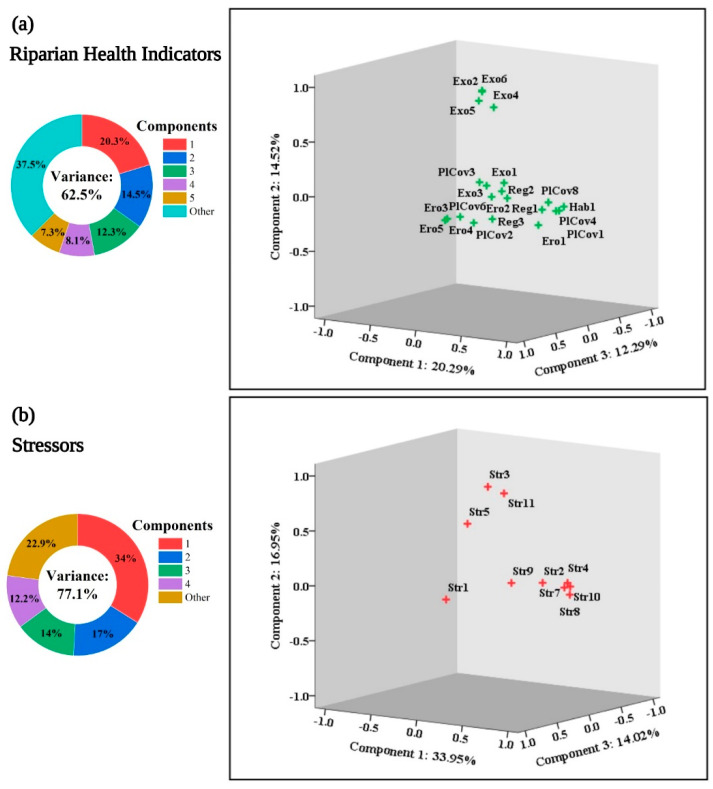
Factor analysis (PCA) plots of riparian health indicators (**a**) and stressors (**b**) for the upper and lower Indus basin riparian zones of the Indus River in Pakistan. The PCA factor loadings of riparian health (**a**) and stressor (**b**) indicator components explain 62.5% and 77.1% of the total variation, respectively. See more details in Figure 2.

The PCA plots for the RHIs and stressors in the IRB are depicted in Figure 4, demonstrating how RHIs and stressor metrics for the UIB and LIB are connected. This study found that variation explained by RHIs varied by 62.50% and stressors by 77.10%. These variations were attributed to the loadings of five and four components, respectively, determined by PCA. In order to ensure the validity of the RHIs and stressor, Kaiser–Meyer–Olkin (0.813 and 0.719) and Bartlett (0.000 for each) sphericity tests were conducted. Additionally, these components were evaluated and validated using two different test methodologies (a screen plot and a Monte Carlo PCA). There were six variables that were significantly associated with the first component of RHIs (PlCov1, Hab1, PlCov4, PlCov8, Reg1, and Ero1). These variables were consistently positive, ranging from 0.914 to 0.707. Similarly, Exo6, Exo2, Exo5, and Exo4 were condensed in the second component and associated positively (0.914 to 0.719). It was found that Ero4 (0.908) responded in a similar way to the other two variables in the third component, Ero5 and Ero3 (0.879 to 0.840), which were also related to each other. The fourth and fifth components consisted of the three (Reg3, PlCov2, and PlCov3) and two (Reg2 and PlCov6) variables, which were all significantly positive (0.777 to 0.491 and 0.837 to 0.770), respectively.

Following the same trends for the stressors, four indicators (Str8, Str10, Str7, and Str4) were shown as the first component and were positively related, ranging from 0.954 to 0.807. Three stressors (Str3, Str11, and Str5) were revealed positively in the second component, ranging from 0.850 to 0.540. A pair of indicators (Str2 and Str1) were grouped together for the third component, with Str2 responding negatively (−0.828). Based on its high value, two indicators (Str4 and Str9) appeared in the fourth component (−0.480 and 0.884).

### 3.3. Relationship between Stressors and Riparian Health Indicators

**Figure 5:**
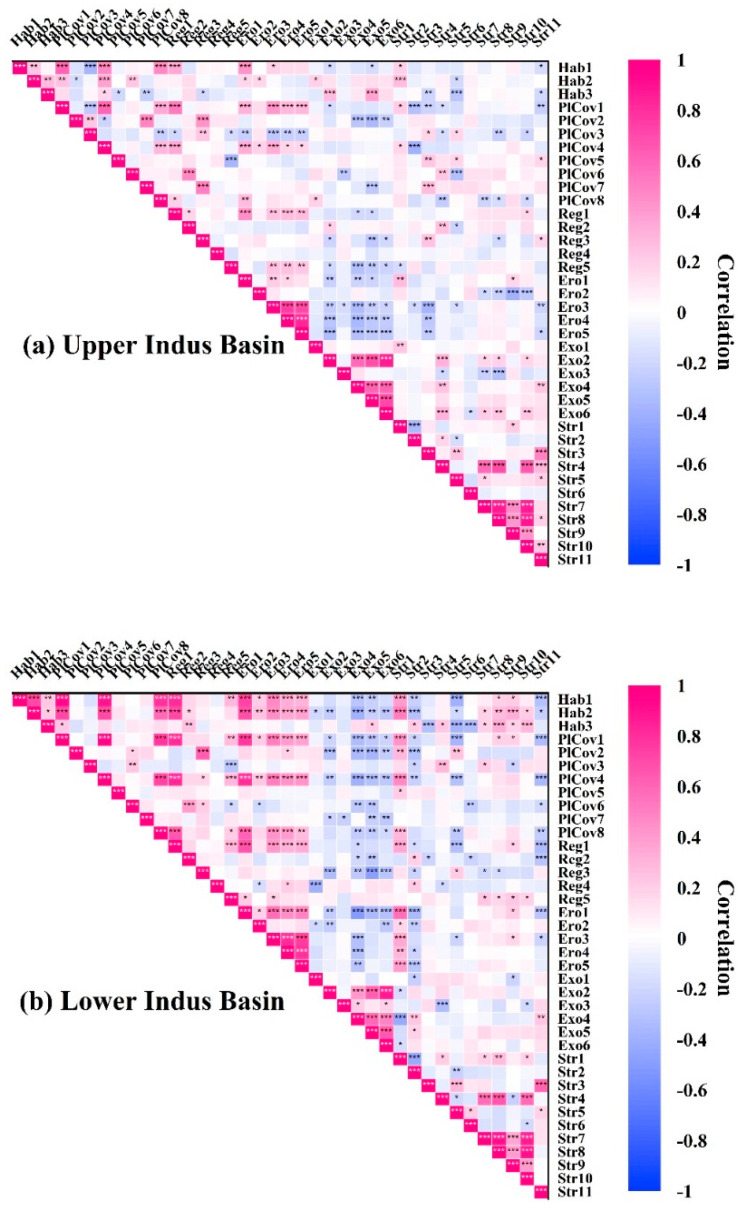
Heat maps of Pearson’s correlation for riparian health indicators with stressors for the upper (**a**) and lower (**b**) Indus basin riparian zones of the Indus River in Pakistan. A dark color indicates a strong relationship, whereas a light color represents a weak relationship. *** Correlation is significant at the 0.001 level (two-tailed); ** Correlation is significant at the 0.01 level (two-tailed); * Correlation is significant at the 0.05 level (two-tailed). See more details in Figure 2.

The Pearson correlation coefficients were used at *p* < 0.001***, *p* < 0.01**, and *p* < 0.05* to determine the relationships between the stressors and RHIs in the riparian zones of the IRB’s UIB and LIB. The indicators of both indices showed positive and negative correlations with moderate to highly significant connections between and among each other (Figure 5). Comparatively, stressors had greater impacts on RHIs in LIB, and they were often associated with higher correlation coefficients in these corridors, ranging from −0.42*** < r < 0.56***. In the LIB, stressors had a strong influence on habitat (−0.39*** < r < 0.50***; Hab1 and Hab2), plant cover (−0.36*** < r < 0.46***; PlCov1 and PlCov4), regeneration (−0.34*** < r < 0.35***; Reg1), erosion (−0.34*** < r < 0.56***; Ero1), and exotics (−0.42*** < r < 0.23**; Exo4). Stressors were significantly correlated among each other in the LIB, ranging from −0.43*** < r < 0.91***; Str1, Str7, and Str8. Similarly, there were strong associations between RHIs in the LIB, with highly correlated values of from −0.51*** < r < 0.94***; Ero1 and PlCov1. Simultaneously, these stressors had a significant impact on the UIB’s habitat (−0.30*** < r < 0.28***; Hab3 and Hab2), plant cover (−0.38*** < r < 0.37***; PlCov4 and PlCov7), regeneration (−0.21* < r < 0.25**; Reg2 and Reg3), erosion (−0.38*** < r < 0.27**; Ero2 and Ero1), and exotics (−0.30*** < r < 0.30**; Exo3 and Exo2) in the UIB.

### 3.4. Comparing Indices and Sub-Indices from the Upper and Lower Indus Basins

**Figure 6:**
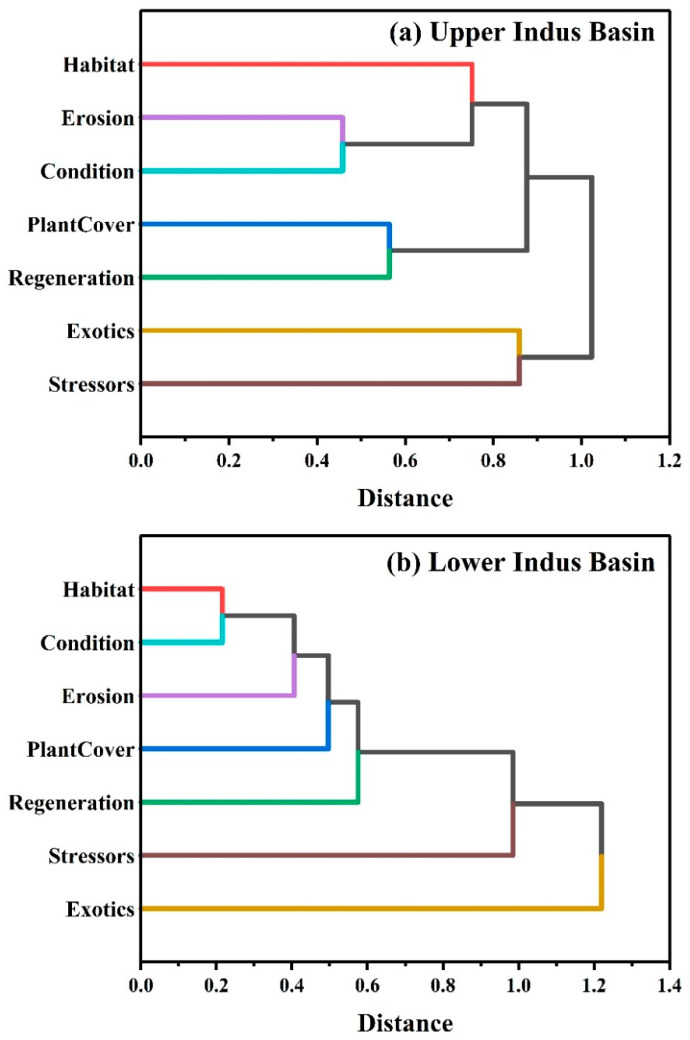
Dendrograms produced by agglomerative hierarchical clustering for riparian health indicators sub-indexes/index with stressors index for the upper (**a**) and lower (**b**) Indus basin riparian zones of the Indus River in Pakistan.

Statistical variations of the UIB and LIB indices and sub-indices were examined in more detail in Figure 6. Using dendrogram plots, the relationships between all of the indices and sub-indices were demonstrated using agglomerative hierarchical clustering, showing the distance between each cluster using the horizontal axis. Seven groups were selected for each situation for both UIB (a) and LIB (b). It was found in the UIB that erosion and condition were clustered together, whereas habitat and condition were first seen together in the LIB. Regeneration and plant cover were grouped together in the second cluster of the UIB, while erosion was identified separately in the LIB. Cluster 3 includes habitat within the UIB and plant cover within the LIB. There was a noticeable difference when exotics and stressors were grouped in the UIB in the fourth cluster, while regeneration, stressors, and exotics appeared differently in the different clusters in the LIB.

## 4. Discussion

The IRB in Pakistan has been exposed to several stressors that have altered its riparian zones. Our results indicate that long-range RHIs and stressors tend to be distributed differently in the UIB and LIB. Similar results have been documented by Arif et al. [2] within the Three Gorges Reservoir, China. In other studies, those indicators have shown different patterns as situations change everywhere [14,36,37], which is consistent with the results obtained in the current study. Changes in riparian zones are influenced by geographical locations and stressors [38,39]. These results indicate that riparian zones in UIB areas are generally better than those in LIB areas (Figure 3). As shown by their relatively high score percentages, all RHIs obtained similar results in the UIB. Although the administrators of the IRB paid equal attention to the UIB and LIB regions, the anthropogenic activities in the LIB zones resulted in greater degrees of stressor impact. In certain locations, the land use system can create a specific stress milieu that alters the environment in specific ways [40,41]. Since UIB possesses a mountainous terrain, riparian areas in the region are less susceptible to stress. Moreover, the Tarbela Dam in Pakistan altered the entire structure of the riparian zone along the Indus River [26]. Species of riparian vegetation present at the study’s time differ from those that existed naturally in the past [29]. It has been recommended that riparian zones with unique characteristics, such as the IRB, be conserved as in other parts of the world [21,42,43]. Other research in other countries, such as Brazil, has also led to similar recommendations [5].

RHIs and stressor indices for the different transects of IUB and LIB were distinguished by Kruskal–Wallis tests (Figure 3). We observed that geographical locations significantly influenced riparian zones in terms of riparian vegetation, as demonstrated by Zema et al. [44]. The statistical analysis in our study was conducted using different methods. River landscapes may be altered because of dam construction in the vicinity [14]. The morphological changes affect the width and circumference of the river, which affect the river’s hydrology [18]. Various factors influence the dynamics of stressors, and hydro-morphological changes are critical to exacerbating their effects [16,45]. When environmental conditions and land use change, these changes may influence the structure and growth of plant communities [36]. Nevertheless, the impact on riparian zones can vary according to the location of the transect and the magnitude of stressors [22]. However, the characteristics of some transects were similar in some respects, so their differences were insignificant. Despite the differences in stressors, the extent of RHIs was very comparable across the study sites. Various land uses may contribute to the differences within a riparian structure. RHIs were always significantly influenced by geographic location for both UIB and LIB. Across the entire IRB area, significant differences were discovered in stressors between transect locations. Furthermore, Zema et al. [44] found significant changes in buffer zones in Italy, considering different environmental conditions and land use patterns. Similar results have been confirmed by multivariate statistical analysis in riparian areas within watersheds. Several studies have used these methods to look at ecological systems and found that they work [15,24,34].

Figure 4 shows similar components for RHIs and stressor indices have been discovered in previous studies [2,32]. Because of their loadings, examining each group separately confirmed that indicators of riparian health and stressors are closely related. Studies have shown that PCA can be applied to a limited number of indicators without requiring wider sets [14,36]. This test likely contributed to the selection of indicators based on the geographical areas within the IRB in terms of contribution and effectiveness. Similar studies on Australian riparian zones have documented the history of selective indicators [23]. Among other indicators, vegetation cover is an effective indicator of riparian health [46,47,48]. Besides being multilayered, it may also serve as a safety filter for other indicators. Although other vegetation characteristics were less affected, the understory cover of riverine vegetation appeared to be more strongly influenced and consequently disappeared in IRB. The flow regulation also affected all RHIs. This study revealed that the riparian health of different sites depends largely on their geographical location, with more drastic changes in the LIB of the IRB and a parallel decline in the UIB parts. Therefore, erosion in the IRB was accelerated, and exotic species were introduced. Similar observations have been observed elsewhere [49,50].

Figure 5 shows the Pearson correlation strength ranges for UIB and LIB. For each river basin, these coefficients indicate their linear strength. The correlation coefficients demonstrated that RHIs change in the IRB. Stressors modify riparian health, and riparian health characteristics vary according to transect location in the IRB. This study highlights the hierarchical relationships between different transects in response to postoperative stressor adjustments resulting from stressor changes. It was found that stressors in these riparian areas might react reversely regardless of the similarity of conditions. Stressor distribution patterns were primarily determined by geographical location. Consequently, we found that the IRB area was fragmented more severely due to this pressure. Because the pattern of distribution was changed, the UIB and LIB have different strengths of Pearson correlation. RHI correlations were highest on transects from LIB areas and relatively low in UIB areas. Perhaps LIB is a relatively plain area with a higher level of anthropogenic activity than UIB. As stressors operate in complex patterns [49,50], our study can assist in assessing the impact of those stressors on riparian health. Several other studies have reached similar conclusions [16,51,52], though some specific riparian zones have reached different conclusions. Cluster analysis of the RHIs and stressors revealed differences in their indices and sub-indices (Figure 6). Published literature indicates that all geographical areas that retain parallel sites are clustered together. Riparian zones underwent significant changes because of human impacts on land-use scenarios [53,54,55,56], which in turn resulted in significant changes in RHIs. Our findings are consistent with those of other studies examining regions such as the IRB.

As a result of the evidence provided above, future research should focus on changes in riparian conditions with topographical features along the banks of main rivers and multiple linked stream systems there. It is intended that these results will provide the river administrator with comprehensive information that will help them to implement functional changes that are appropriate to the condition of the respective water body.

## 5. Conclusions

This study comprehensively demonstrates how stressors affect riparian zones in large river systems. Furthermore, it highlights the management strategies that are needed to protect the environment from such stressors. There is currently no general buffer protection regulation for river systems in river-dependent agricultural countries such as Pakistan. From an ecological and environmental perspective, there are many advantages associated with riparian buffers. We found that the riparian zones in the Indus River basin could not offset the negative effects of stressors. This meant that national remediation efforts were required to protect these water sources. Riparian buffers are inadequate to maintain the required functionality of river systems on their own. Our assessment indicated that the upper Indus basin is significantly more functional regarding habitat, vegetable cover, regeneration, exotics, and erosion indicators. Nevertheless, the indices of RHIs and stressors varied significantly across the lower Indus basin as well. We identified the key RHIs and stressors based on principal component analysis, which adequately explained the variance for both indices. Stressors in LIB had more significant impacts on RHIs as determined by the Pearson correlation. Moreover, our results indicated that stressors affected RHI indices in different ways for habitat, plant cover, regeneration, exotics, and erosion indicators. It was also confirmed by the agglomerative hierarchical cluster that the RHI and stressor indices and sub-indices differed by UIB and LIB. People who run similar large rivers or provide ecosystem services to people and want to reduce the damage that stressors produce to the environment may find the results of this study useful.

## Data Availability

The data that support the findings of this study are available from the corresponding author upon reasonable request.

## Supplementary Materials

The following supporting information can be downloaded at: https://www.mdpi.com/article/10.3390/ijerph192013239/s1.
