# Supplementary material for: Impacts of Stressors on Riparian Health Indicators in the Upper and Lower Indus River Basins in Pakistan
Source: Int J Environ Res Public Health. 2022 Oct 14;19(20):13239. doi: 10.3390/ijerph192013239 (PMC9603529; doi:10.3390/ijerph192013239)
Supplement: Supplementary file 1 [file ijerph-19-13239-s001.zip › ijerph-1940184-supplementary.pdf]

Supplementary Material

Impacts of Stressors on Riparian Health Indicators in the Upper and Lower Indus River Basins in Pakistan

Amin Hira [1,†], Muhammad Arif [2,*,†], Nowsherwan Zarif [3], Zarmina Gul [4], Xiangyue Liu [1] and Yukun Cao [1,*]

[1] Department of Forestry Economics & Management, Northeast Forestry University, Harbin 150040, China
[2] Biological Science Research Center, Academy for Advanced Interdisciplinary Studies, Southwest University, Chongqing 400715, China
[3] Pakistan Forest Institute, Peshawar 25000, Pakistan
[4] College of Chemistry, Chemical Engineering and Resource Utilization, Northeast Forestry University, Harbin 150040, China
* Correspondence: muhammadarif@swu.edu.cn (M.A.); cyklk@163.com (Y.C.)
† These authors contributed equally to this work.

Supplementary Materials for field investigation

As mentioned in the **field investigation** (**2.2**), **lines 156-170**: *The aim of this study was to obtain real-life data on RHIs and stressors by adapting an innovative approach from the literature (see Arif et al. [2,15]). The data collection methods for each parameter are described in a variety of sources (e.g., literature, re-ports, and databases). This approach was introduced by Jansen et al. [33] to study the riparian zone in Australia, and since then it has been adopted by other researchers [19,23,34]. This study included several indicators that were subject to constraints spe-cific to the IRB. Thus, we used the method described by Lanzanova et al. [34] to esti-mate new indicators and to fill in missing measurements. Each component of the ri-parian zone was quantified by establishing a 100-meter-long, 20-meter-wide transect parallel to the Indus River (see Supplementary Materials for more information). Indi-cators of the riparian zone were studied from two perspectives (see Figure 2). Indica-tors such as habitat, plant cover, regeneration, exotics, and erosion are included in the RHI category. The indicators shown in this study corresponded to the actual indicators observed in riparian zones during the study period. The second category consists of broad stressors.*

2. Arif, M.; Jie, Z.; Tahir, M.; Xin, H.; Changxiao, L. The impact of stress factors on riparian and drawdown zones degradation around dams and reservoirs. *Land Degrad. Dev.* **2022**, 2127-2147. https://doi.org/10.1002/ldr.4310.
15. Arif, M.; Behzad, H.M.; Tahir, M.; Li, C. The impact of ecotourism on ecosystem functioning along main rivers and tributaries: Implications for management and policy changes. *J. Environ. Manage.* **2022**, *320*, 115849. https://doi.org/10.1016/j.jenvman.2022.115849.
19. Muhammad, A.; Behzad, H.M.; Tahir, M.; Changxiao, L. Environmental literacy affects riparian clean production near major waterways and tributaries. *Sci. Total Environ.* **2022**, *834*, 155476. https://doi.org/10.1016/j.scitotenv.2022.155476.
23. Johansen, K.; Phinn, S.; Dixon, I.; Douglas, M.; Lowry, J. Comparison of image and rapid field assessments of riparian zone condition in Australian tropical savannas. *For. Ecol. Manag.* **2007**, *240*, 42-60. https://doi.org/10.1016/j.foreco.2006.12.015.
33. Jansen, A.; Robertson, A.; Leigh, T.; Andrea, W. *Rapid appraisal of riparian condition*; 2005; Volume 4A, p. 16p.
34. Muhammad, A.; Behzad, H.M.; Tahir, M.; Changxiao, L. Nature-based tourism influences ecosystem functioning along waterways: Implications for conservation and management. *Sci. Total Environ.* **2022**, *842*, 156935. https://doi.org/10.1016/j.scitotenv.2022.156935.

Here are some pages relating to the research method by describing the gradual development and history of the method we used in our manuscript.

1. The validity of the methodology used in this study.

Figure S1. Officially, this approach is used in Australia.

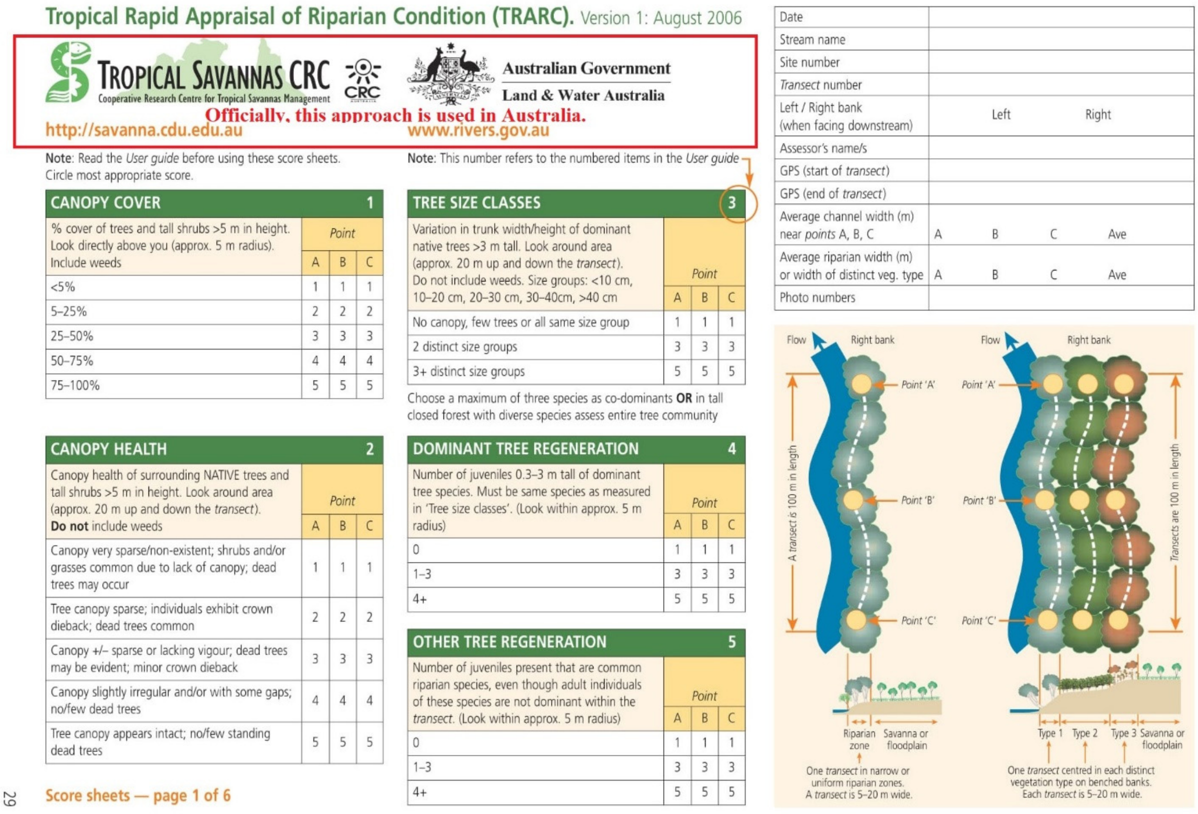

Tropical Rapid Appraisal of Riparian Condition (TRARC). Version 1: August 2006

TROPICAL SAVANNAS CRC
Cooperative Research Centre for Tropical Savannas Management
Australian Government
Land & Water Australia
Officially, this approach is used in Australia.
http://savanna.cdu.edu.au
www.rivers.gov.au

Note: Read the *User guide* before using these score sheets. Circle most appropriate score.

Note: This number refers to the numbered items in the *User guide*

CANOPY COVER 1			
% cover of trees and tall shrubs >5 m in height. Look directly above you (approx. 5 m radius). Include weeds	Point A	Point B	Point C
<5%	1	1	1
5–25%	2	2	2
25–50%	3	3	3
50–75%	4	4	4
75–100%	5	5	5

CANOPY HEALTH 2			
Canopy health of surrounding NATIVE trees and tall shrubs >5 m in height. Look around area (approx. 20 m up and down the *transect*). **Do not** include weeds	Point A	Point B	Point C
Canopy very sparse/non-existent; shrubs and/or grasses common due to lack of canopy; dead trees may occur	1	1	1
Tree canopy sparse; individuals exhibit crown dieback; dead trees common	2	2	2
Canopy +/– sparse or lacking vigour; dead trees may be evident; minor crown dieback	3	3	3
Canopy slightly irregular and/or with some gaps; no/few dead trees	4	4	4
Tree canopy appears intact; no/few standing dead trees	5	5	5

TREE SIZE CLASSES 3			
Variation in trunk width/height of dominant native trees >3 m tall. Look around area (approx. 20 m up and down the *transect*). Do not include weeds. Size groups: <10 cm, 10–20 cm, 20–30 cm, 30–40cm, >40 cm	Point A	Point B	Point C
No canopy, few trees or all same size group	1	1	1
2 distinct size groups	3	3	3
3+ distinct size groups	5	5	5

Choose a maximum of three species as co-dominants **OR** in tall closed forest with diverse species assess entire tree community

DOMINANT TREE REGENERATION 4			
Number of juveniles 0.3–3 m tall of dominant tree species. Must be same species as measured in 'Tree size classes'. (Look within approx. 5 m radius)	Point A	Point B	Point C
0	1	1	1
1–3	3	3	3
4+	5	5	5

OTHER TREE REGENERATION 5			
Number of juveniles present that are common riparian species, even though adult individuals of these species are not dominant within the *transect*. (Look within approx. 5 m radius)	Point A	Point B	Point C
0	1	1	1
1–3	3	3	3
4+	5	5	5

Date				
Stream name				
Site number				
Transect number				
Left / Right bank (when facing downstream)	Left		Right	
Assessor's name/s				
GPS (start of *transect*)				
GPS (end of *transect*)				
Average channel width (m) near *points* A, B, C	A	B	C	Ave
Average riparian width (m) or width of distinct veg. type	A	B	C	Ave
Photo numbers				

29 Score sheets — page 1 of 6

Figure S2. An example of how to make a transect on the sample site.

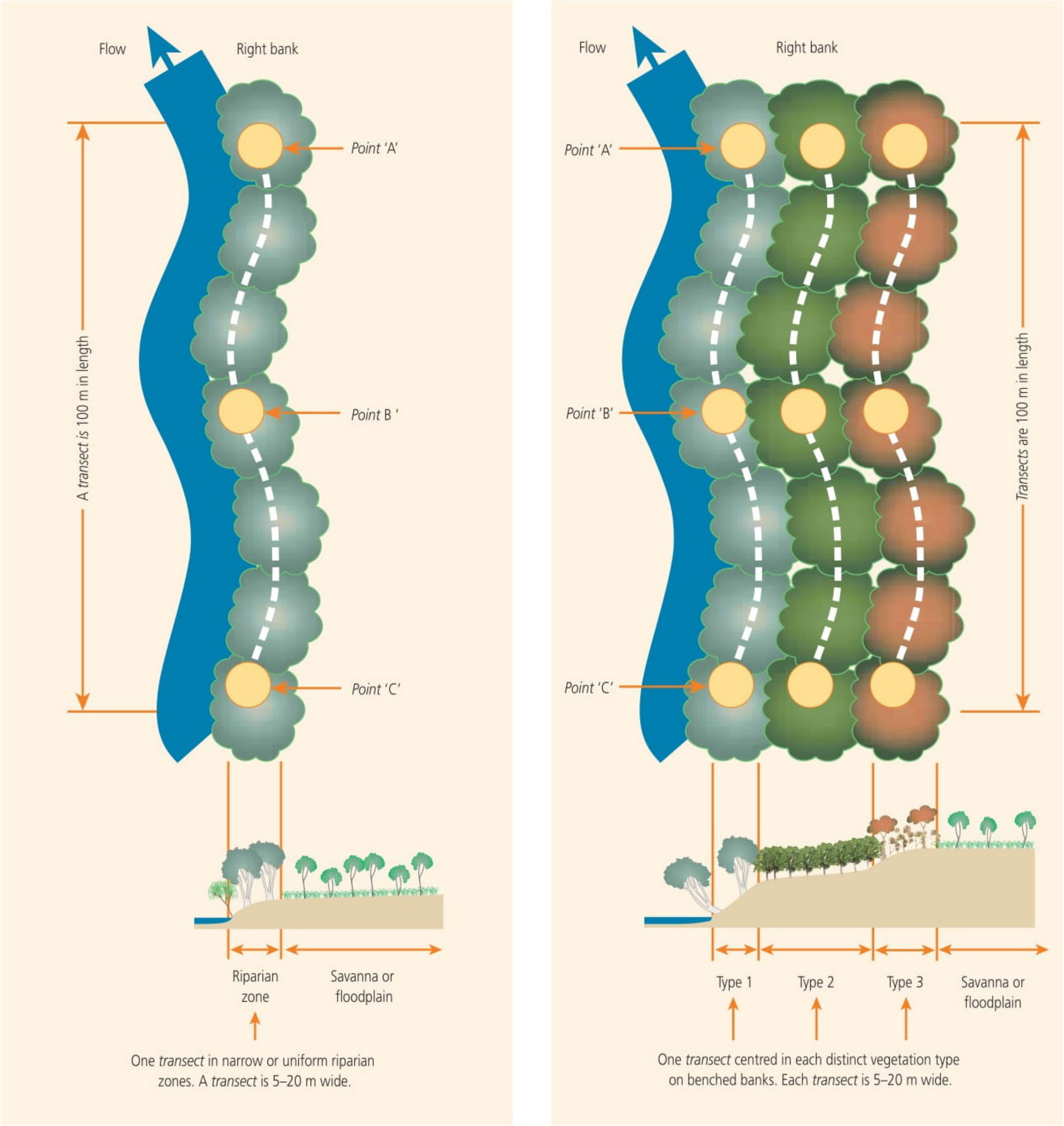

Figure S3. How to measure the tree cover or canopy cover within the transect.

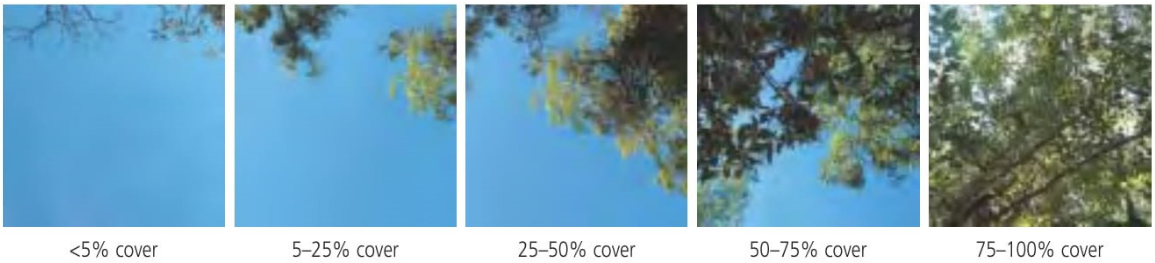

Figure 8. Examples of five 'Canopy cover' categories.

Figure S4. How to measure the tree regeneration within the transect.

4. Dominant tree regeneration

Are there juveniles (<3 m tall) of the dominant tree species?

Assess at the three *points* along the *transect*. When standing at *points* A, B and C, select the same species as scored above in 'Tree size classes' and count the number of juvenile plants (0.3–3 m tall) around you (approximately a 5 m radius, Figure 11). Coppicing (regrowth from fallen trees or stumps) can be included as juveniles if they are <3 m tall.

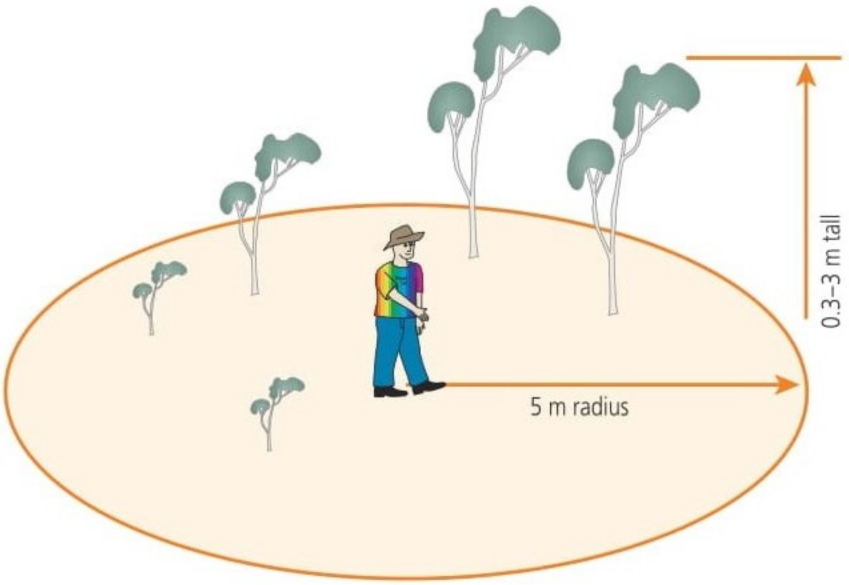

Figure 11. Juvenile plants (0.3–3 m tall) are counted within a 5 m radius area.

Figure S5. How to measure the grass or forb cover within the transect.

Figure 13. Example of how to assess percentage cover within a 5 x 5 metre square.

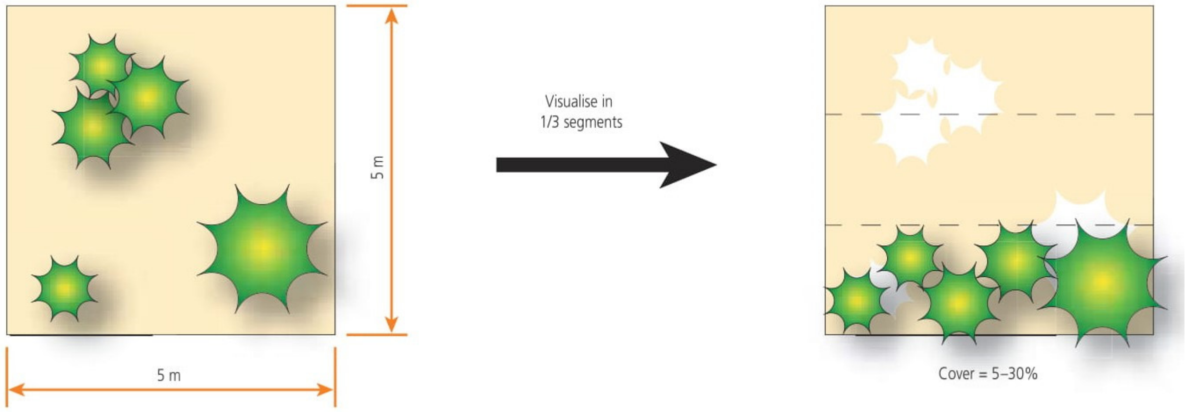

Figure S6. How to measure the slumping, gullying, and undercutting within the transect.

18. Slumping, gullying and undercutting

How much of the river bank has eroded due to slumping, gullying and undercutting?

Assess along the *transect* (100 m long, 5–20 m wide). For each erosion feature, estimate the combined width of slumps, active gullies and undercutting within the *transect* (Figure 28 and Figure 29). Separate scores are given to each erosion feature. You may also wish to note the height and depth of each erosion feature (these are not included in the score). Active gullies are unstable and may be increasing in size. Stable or natural gullies may have vegetation, rocks or other structures supporting their walls and head (top of gully).

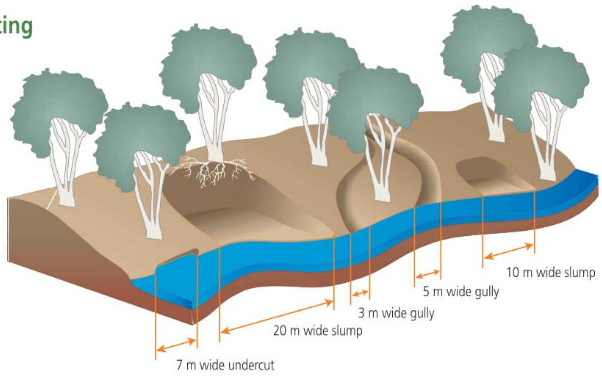

Figure 28. Combined widths of each erosion feature (measured within the 100 m long x 5–20 m wide *transect*): slumping = 30 m; gullying = 8 m; and undercutting = 7 m. A score is given to each of these erosion features.

Figure 29. Examples of the three erosion features measured in the TRARC: slumping of small bank (top left); slumping of large bank (top right); active (unstable) gully (bottom left); and, undercutting (bottom right).

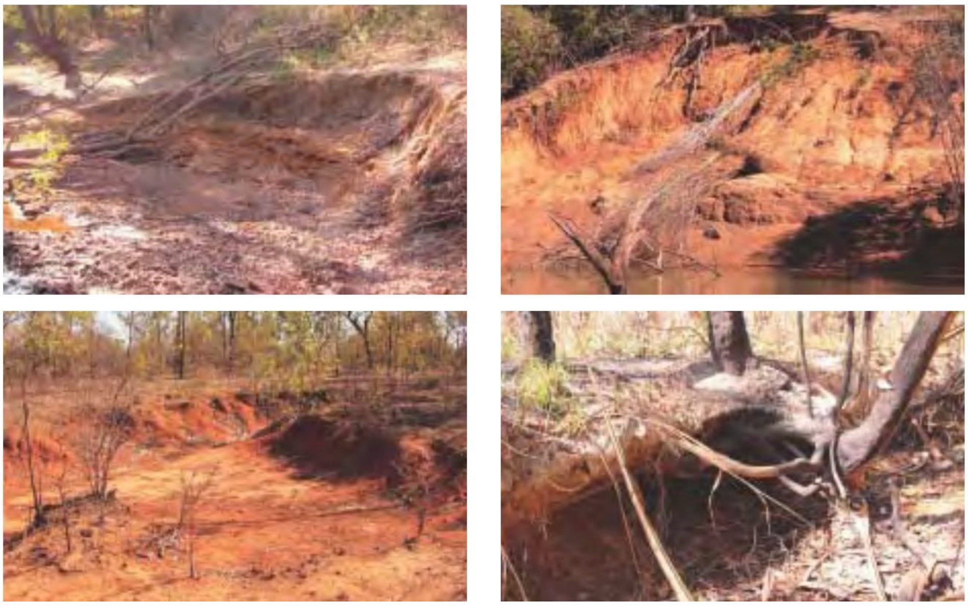

We have provided visual examples of our methodology and have quoted some indicators from our study.

<u>Please feel free to ask any additional questions regarding the materials and methods.</u>

<u>Dr. Muhammad Arif; Email: muhammadarif@swu.edu.cn</u>
